# Knowledge regarding extracorporeal membrane oxygenation management among Brazilian pediatric intensivists: a cross-sectional survey

**DOI:** 10.5935/2965-2774.20230350-en

**Published:** 2023

**Authors:** José Colleti Júnior, Arnaldo Prata-Barbosa, Orlei Ribeiro Araujo, Cristian Tedesco Tonial, Felipe Rezende Caino de Oliveira, Daniela Carla de Souza, Fernanda Lima-Setta, Thiago Silveira Jannuzzi de Oliveira, Mary Lucy Ferraz Maia Fiuza de Mello, Carolina Amoretti, Paulo Ramos David João, Cinara Carneiro Neves, Norma Suely Oliveira, Cira Ferreira Antunes Costa, Daniel Garros

**Affiliations:** 1 Department of Pediatrics, Hospital Israelita Albert Einstein - São Paulo (SP), Brazil; 2 Instituto D’Or de Pesquisa e Ensino - Rio de Janeiro (RJ), Brazil; 3 Grupo de Apoio ao Adolescente e à Criança com Câncer, Instituto de Oncologia Pediátrica, Universidade Federal de São Paulo - São Paulo (SP), Brazil; 4 Department of Pediatrics, Universidade Federal do Rio Grande do Sul - Porto Alegre (RS), Brazil; 5 Department of Pediatrics, Hospital Alvorada Moema - São Paulo (SP), Brazil; 6 Department of Pediatrics, Hospital Universitário, Universidade de São Paulo - São Paulo (SP), Brazil; 7 Department of Pediatrics, Neocenter - Hospital Felício Rocho - Belo Horizonte (MG), Brazil; 8 Department of Pediatrics, Santa Casa do Pará - Belém (PA), Brazil; 9 Department of Pediatrics, Hospital Universitário Professor Edgar Santos, Universidade Federal da Bahia - Salvador (BA), Brazil; 10 Department of Pediatrics, Hospital Pequeno Príncipe - Curitiba (PR), Brazil; 11 Department of Pediatrics, Hospital Infantil Albert Sabin - Fortaleza (CE), Brazil; 12 Department of Pediatrics, Universidade Federal do Espírito Santo - Vitória (ES), Brazil; 13 Department of Pediatrics, Hospital Materno Infantil de Brasília - Brasília (DF), Brazil; 14 Stollery Childrens Hospital, University of Alberta - Edmonton, Canada

**Keywords:** Extracorporeal membrane oxygenation, Survey and questionnaires, Health knowledge, attitudes, practice, Child, Pediatric intensive care units

## Abstract

**Objective:**

To assess Brazilian pediatric intensivists’ general knowledge of extracorporeal membrane oxygenation, including evidence for its use, the national funding model, indications, and complications.

**Methods:**

This was a multicenter cross-sectional survey including 45 Brazilian pediatric intensive care units. A convenience sample of 654 intensivists was surveyed regarding their knowledge on managing patients on extracorporeal membrane oxygenation, its indications, complications, funding, and literature evidence.

**Results:**

The survey addressed questions regarding the knowledge and experience of pediatric intensivists with extracorporeal membrane oxygenation, including two clinical cases and 6 optional questions about the management of patients on extracorporeal membrane oxygenation. Of the 45 invited centers, 42 (91%) participated in the study, and 412 of 654 (63%) pediatric intensivists responded to the survey. Most pediatric intensive care units were from the Southeast region of Brazil (59.5%), and private/for-profit hospitals represented 28.6% of the participating centers. The average age of respondents was 41.4 (standard deviation 9.1) years, and the majority (77%) were women. Only 12.4% of respondents had taken an extracorporeal membrane oxygenation course. Only 19% of surveyed hospitals have an extracorporeal membrane oxygenation program, and only 27% of intensivists reported having already managed patients on extracorporeal membrane oxygenation. Specific extracorporeal membrane oxygenation management questions were responded to by only 64 physicians (15.5%), who had a fair/good correct response rate (median 63.4%; range 32.8% to 91.9%).

**Conclusion:**

Most Brazilian pediatric intensivists demonstrated limited knowledge regarding extracorporeal membrane oxygenation, including its indications and complications. Extracorporeal membrane oxygenation is not yet widely available in Brazil, with few intensivists prepared to manage patients on extracorporeal membrane oxygenation and even fewer intensivists recognizing when to refer patients to extracorporeal membrane oxygenation centers.

## INTRODUCTION

Extracorporeal membrane oxygenation (ECMO) is a lifesaving rescue tool for refractory respiratory and/or circulatory failure and is a major component of extracorporeal life support (ECLS) programs.^(1)^ There are fundamental differences in pediatric ECMO patients compared to adults, including indications, circuit setup, sites of cannulation, and techniques.^(2,3)^ The use of ECMO in pediatrics is increasing, and the Extracorporeal Life Support Organization (ELSO) reported that 23.2% of all ECMO runs performed in the last 5 years were in children and neonates.^(4)^

The role of ECMO within the pediatric intensive care unit (ICU) technological array has been growing, and survival has been increasing over the last few decades.^(3)^ Extracorporeal membrane oxygenation has been recognized as rescue therapy for severe respiratory and/or cardiac failure, bridging patients to a decision, to recovery, or to transplantation for both lungs and hearts.^(5,6)^ In cardiac patients, ECMO can also be a bridge to another form of circulatory mechanical support, such as ventricle-assisted devices.^(7,8)^

Despite the worldwide increase in ECMO runs, currently, there are only 26 ELSO-certified ECMO centers in Brazil, which results in few physicians with sufficient experience in this technology.^(9)^ Importantly, it is unknown if general pediatric intensivists are aware of the scientific evidence and the most common indications, complications, and other particularities of ECMO, knowledge that is fundamental for proper and timely referral in a large country such as Brazil. Thus, this study ascertains the overall knowledge of a large sample of Brazilian pediatric intensivists regarding the role of ECMO in severe respiratory and cardiac failure.

## METHODS

This study was conducted using a survey of Brazilian pediatric intensivists and was approved by the Institutional Review Board of *Hospital Assunção Rede D’Or* (CAAE 46174521.9.0000.5625). The participating centers were recruited from the Brazilian Research Network in Pediatric Intensive Care (BRnet-PIC) database.^(10)^ The centers invited to participate were conveniently chosen from each Brazilian state, in proportion to the state’s population, to gather a representative sample of pediatric ICUs in Brazil. It is important to recognize that Brazilian intensivists usually work in more than one pediatric ICU. We asked them to respond to the survey as independent practitioners and inform the hospital where they spend most of their time.

The instrument was tested according to the methodology of Burns et al.,^(11)^ and 5 experts in the field gave feedback on content and structure. Their suggestions were analyzed and incorporated into the final version.

A preliminary exploratory survey was distributed in August 2021 to the pediatric ICU chiefs and department heads of 45 hospitals in Brazil, whose contacts were obtained through BRnet-PIC. The objective was to obtain information about the characteristics of their units regarding the number of beds, types of patients admitted (mixed medical and surgical including cardiac and noncardiac patients or exclusively cardiac patients), staff numbers, and their willingness to participate in the study.

The main survey was then distributed using a link via *WhatsApp* on November 16, 2021, to all pediatric intensivists of the participating centers and remained open for 1 mo. A weekly reminder was sent to everyone via a national pediatric ICU network that uses WhatsApp. The survey was anonymous and was recorded in REDCap (Vanderbilt, Nashville, USA).^(12)^ The second part of the main survey included two clinical scenarios (a respiratory failure case and a cardiogenic shock case secondary to myocarditis); the subject was questioned whether ECMO would be indicated as a form of support. The last section of the survey was optional and invited pediatric ICU physicians who had experience with patients on ECMO to answer further technical questions. Subjects who had not managed ECMO patients before could end the survey without prejudice. This subsection included more questions about the previously described clinical cases, with detailed management questions that would require ECMO training and specific knowledge. The questions were obtained and adapted from an ECMO training course that has been frequently administered in Brazil (personal files, D.G.).

Data were quantified using descriptive statistics. The analysis and graphs were performed using the software R (version 4.0.1, The R Foundation for Statistical Computing, Vienna, Austria).^(13)^

## RESULTS

Overall, 45 Brazilian pediatric ICUs initially agreed to participate in the study. The first survey (exploratory) was effectively completed by 42 centers (91%) that employed 654 pediatric intensivists. The main survey (ECMO knowledge) was completed by 63% (412/654) of pediatric intensivists. Most pediatric ICUs were from the Southeast region of Brazil (59.5%), which has 42.2% of the Brazilian population and the highest concentration of pediatric ICUs in the country. The characteristics of the respondents and participating centers are shown in table 1. Private hospitals represented 28.6% (12/42) of the participating centers, followed by public nonacademic hospitals (26.2%, 11/42). The median number of hospital beds was 250 (interquartile range - IQR 146.2 - 400), and the median number of pediatric ICU beds was 10 (IQR 8-18). Most pediatric ICUs (61.9%) admit only pediatric patients (not newborns) and admit both clinical and surgical patients (88.1%). According to the exploratory survey, only 19% (8/42) of the participating pediatric ICUs had an ECMO program at their institution.

**Table 1:** Characteristics of participants

Variable	
Sex (female)	275 (77.2)
Age (years)	41.5 ± 9.1
Experience in pediatric critical care (years)	13.1 ± 9.3
Board-certified in pediatric critical care	185(52%)
ECMO experience	
ECMO course	44(12.4)
Managed patient(s) on ECMO	93 (26.6)
No ECMO available in hospital	199(56.9)
Type of hospital	
Public (general)	11 (26.2)
Public (academic/university)	8(19.0)
Philanthropic	7(16.7)
Private	12(28.6)
Other	4 (9.5)
Beds	
Hospital beds	250(146.2-400)
Pediatric beds	55.5(21 -87.7)
PICU beds	10(8-18)
Type of pediatric ICU	
Pediatric patients exclusively	26(61.9)
Mixed (pediatric and neonatal) patients	15(35.7)
Other	1 (2.4)
Pediatric ICU patients	
Clinical patients only	1 (2.4)
Clinical and surgical patients	37(87.1)
Cardiac patients only (clinical and surgical)	1 (2.4)
Oncologic patients only	1 (2.4)
Other	2 (4.8)

ECMO - extracorporeal membrane oxygenation; ICU - intensive care unit. Results expressed as n (%), mean ± standard deviation or median (interquartile range 25 - 75).

The most questions were not mandatory; hence, the denominator varied according to the response rate.

The mean age of respondents was 41.5 years (standard deviation - SD 9.1), and most were women (77.2%). The mean time spent working in a pediatric ICU was 13.1 years (SD 9.3). A majority (97%) had pediatric residency training, and 75% had subspecialty training in pediatric intensive care; 52% were board-certified in pediatric intensive care. Only 12.4% (44/359) had taken an ECMO course, according to their own definition of such training. In 25.1% (88/350) of the responses, intensivists reported that their hospital offered ECMO for adults and children. In 6.9% (24/350), ECMO was available only for adults, and in another 7.4% (26/350), ECMO was available only for children. Only 26.6% (93/350) of respondents reported having managed patients on ECMO; 60.2% (56/93) of them reported having treated between 2 and 5 patients.

**Figure 1:**
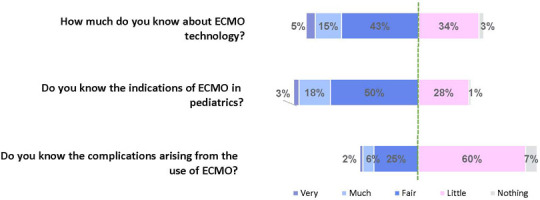
The percentages of responses showing knowledge about extracorporeal membrane oxygenation, its indications, and complications (Likert scale)

Most subjects (62.3%, 218/350) reported thinking about ECMO as rescue therapy for patients with severe acute respiratory failure when standard therapies have failed, while 31.1% (109/350) reported not considering it because this therapy is not available for them. Knowledge about indications and complications is shown in figure 1. Although 71% responded that they know fair to very much about the indications for ECMO, 67% responded that they know little/nothing about complications when using ECMO.

The green line separates the most divergent groups. ECMO - extracorporeal membrane oxygenation.

We also asked whether they believed there is sufficient scientific evidence for the use of ECMO as rescue therapy for pediatric patients with severe acute respiratory failure, and *64%* (225/300) responded positively. The reasons are depicted in Supplementary Material - Table 1S.

We also asked questions regarding funding. Of note, 38% (70/185) of respondents stated that, according to their knowledge, ECMO was funded by private health insurance, followed by the Public National Health System (SUS - *Sistema Único de Saúde)* with 32% (60/185). When asked “Ideally, how do you think it should be funded?”, 67.1% (233/347) responded that it should be funded by the SUS and 46.7% (165/347) that it should be funded by private health insurance. Only 5.2% (18/347) responded that it should be paid for out of pocket (patient/family). The full array of questions regarding ECMO funding is shown in Supplementary Material - Table 2S.

### Clinical case 1

**Figure 2:**
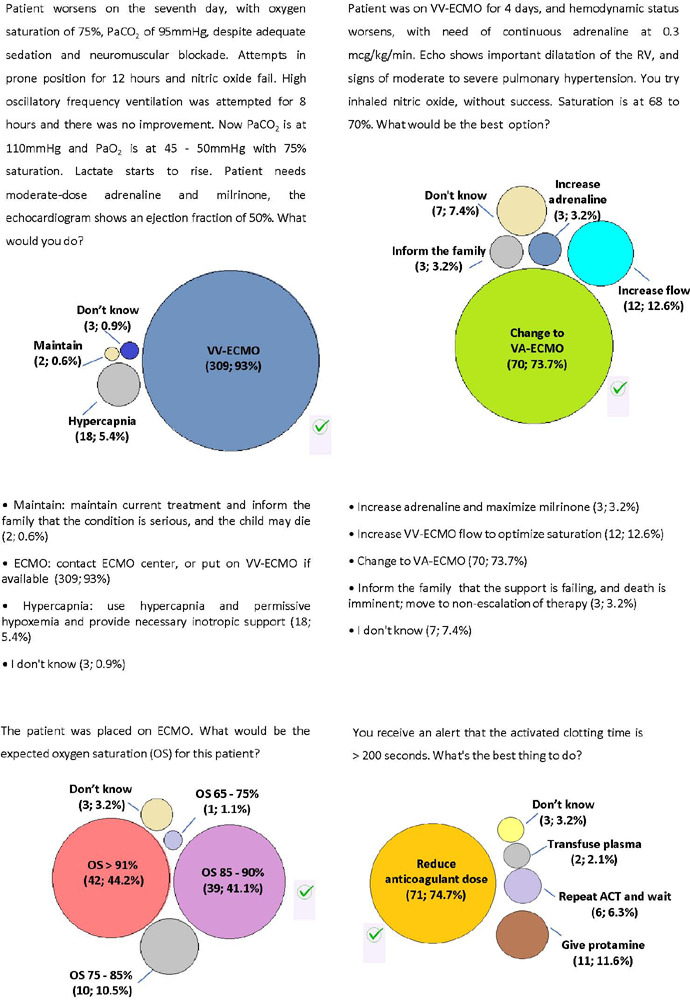
Clinical case 1 : questions and answers of the respondents

A 12-year-old male patient weighing 40kg, was admitted to the pediatric ICU due to severe community-acquired pneumonia. He was intubated and started conventional mechanical ventilation (MV) on the second day of admission. Figure 2 shows the questions and responses.

PaCO_2_ - partial pressure of carbon dioxide; PaO_2_ - partial pressure of oxygen; ECMO - extracorporeal membrane oxygenation; W - venovenous; VA - venoarterial; OS - oxygen saturarion; ACT - activated clotting time.

### Clinical case 2

**Figure 3:**
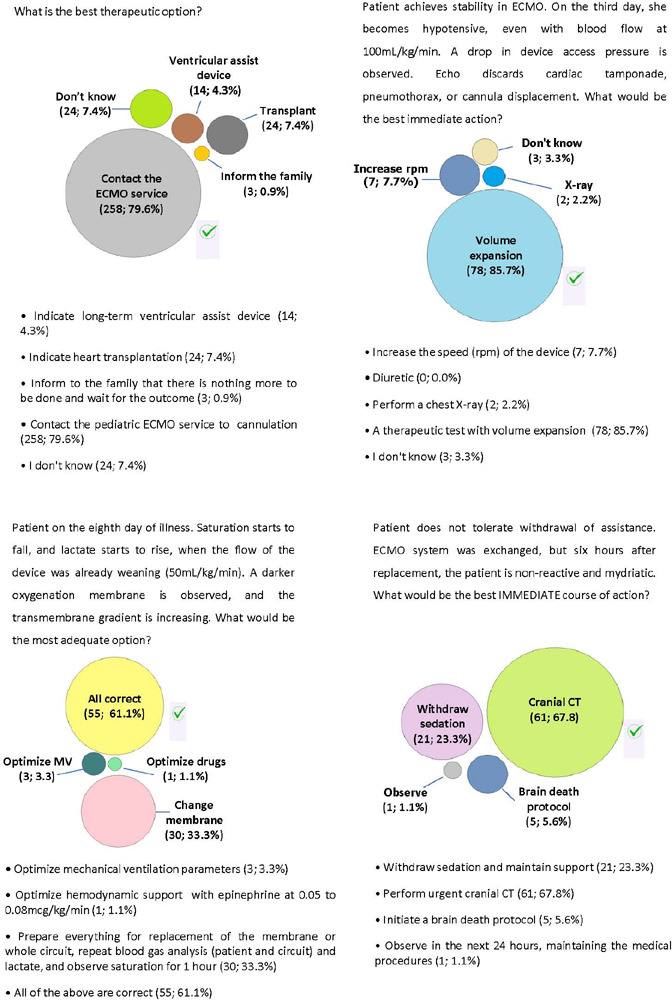
Clinical case 2: questions and answers of the respondents

A 9-month-old female weighing 7kg was admitted to the pediatric ICU for 48 hours with viral myocarditis. She was on invasive MV, and the echocardiogram showed an ejection fraction of 20%. She now receives vasoactive drugs at very high levels, and her hemodynamic status is deteriorating. The mean arterial pressure is now in the 15th percentile. She had already received fluid resuscitation, and an attempt with levosimendan had failed. Figure 3 shows the questions and responses.

ECMO - extracorporeal membrane oxygenation; MV - mechanical ventilation; CT - computed tomography.

When asked if they wanted to answer specific questions about ECMO management, only 15.5% (64/412) answered, corresponding approximately to the number of intensivists who have ECMO at their center. The questions and answers are shown in table 2. The overall rate of correct answers was fair/good (median 63.4%, ranging from 32.8% to 91.9%).

**Table 2:** Correct answers to the specific questions about extracorporeal membrane oxygenation management

**These are signs of membrane oxygenator failure, except**	
Decreased transfer of oxygen and carbon dioxide	3, 4.7%
Blood in the gas phase of the oxygenator	12, 18.8%
Increased pre- and postoxygenator pressure	36, 56.3%*
Increase in preoxygenator pressure	6, 9.4%
Increased hemolysis	7, 10.9%
**When isolating the patient from ECMO, which tube is clamped first?**	
Venous	16, 24.6%
Arterial	37, 56.9%*
Both together	12, 18.5%
**On VA-ECMO, a patient’s Path approaching the postoxygenator PaO_2_ indicates:**
Better oxygen delivery	14, 21.9%
Improved membrane function	9, 14.1%
Improved native lung function	20, 31.3%
Decreased native cardiac output	21, 32.8%*
Anemia	0
**On VA-ECMO, a normal acceptable venous saturation range is:**	
**50 - 60%**	2, 3.1%
**65 - 75%**	50, 78.1%*
**80 - 90%**	8, 12.5%
**> 90%**	4, 6.3%
**The following factors influence the provision of oxygen in W-ECMO**
Pump flow rate	14, 22.6%
Hemoglobin saturation	13, 21.0%
Cardiac output	12, 19.4%
Recirculation	13, 21.0%
All above are correct	57, 91.9%*
**Factors that influence the provision of oxygen in VA-ECMO (select all that apply)**	
Pump flow rate	55, 85.9%*
Native cardiac output	27, 42.2%*
Recirculation	28, 43.8%
Sweep gas flow rate	26, 40.6%
Postoxygenator pressure	35, 54.7%

ECMO - extracorporeal membrane oxygenation; VA - venoarterial; PaO_2_ - partial pressure of oxygen; W-venovenous. * Correct answers. Results expressed as n (%),

## DISCUSSION

We demonstrated in this study that a minority of a representative sample of Brazilian pediatric intensivists had been exposed to ECMO management, and most participants had limited knowledge of the role of ECMO in respiratory and cardiac failure. Approximately one-fifth of all subjects reported having some experience with ECMO. Of the respondents who self-reported having familiarity with ECMO, the majority performed fairly, both in the clinical cases and in the specific technical questions.

Interestingly, although 21% reported knowing much/ very much about the indications for ECMO in pediatric patients, only 8% reported knowing much/very much about ECMO complications. This has significant implications, especially in the informed consent process with families. The attending physician is expected to understand the mechanical and clinical complications of this advanced therapy to properly inform the families of critically ill children to whom this form of support may be offered.

There is a paucity of studies in the medical literature addressing physicians’ knowledge of ECMO. Uezato et al. surveyed medical students regarding their understanding of the role of ECMO in COVID-19 patients after a cycle of lectures and concluded that the teaching managed to raise the students’ knowledge.^(14)^

Extracorporeal membrane oxygenation has been available in Brazilian pediatric ICUs since the mid-1990s.^(15)^ Currently, there is no standardized certification process for an ECMO specialist in the country. ELSO has well-organized educational modules to train clinicians and has established specific guidelines for developing and maintaining ECMO programs around the world.^(16)^ There is an active South American ELSO chapter, and many programs in Brazil are now established as registered centers.^(4)^ The ELSO guidelines provide a structure for each ECMO center to develop its institution-specific practices and policies according to minimal standards. However, any institution can have an ECMO program without being a member of ELSO, and there is no official requirement by any regulatory authority for minimal standards for training and qualifications in Brazil. ECMO education programs, both theoretical and practical with advanced simulation, should be strongly recommended by intensive care societies as a minimal requirement in countries where ECMO knowledge is incipient, such as Brazil.^(17,18)^ In fact, Miana et al. published evidence of the positive impact of organized ECMO training on the outcome of cardiac patients in Brazil.^(19)^

Sixty-four percent of the respondents believed that there is sufficient scientific evidence for the use of ECMO as rescue therapy for pediatric patients with severe acute respiratory failure, and most of them (67.6%) said that there is high-quality evidence in the medical literature supporting ECMO for those patients. However, most evidence comes from studies in adult patients, while ECMO in pediatrics remains somewhat controversial. No randomized controlled trials have been conducted to date to test ECMO as an intervention in pediatric patients with a critical illness.^(20)^ However, for some specific clinical conditions, there is some evidence of its value. A systematic review and meta-analysis on the role of ECMO in children with refractory septic shock, despite its inherent limitations, concluded that there is enough evidence to recommend ECMO for all pediatric age groups.^(21)^ For cardiac patients postcardiotomy and with myocarditis/cardiomyopathy, there is evidence that ECMO improves survival based on large database review studies.^(22,23,24)^ Regarding neonates, the use of ECMO is supported by three clinical trials.^(25,26,27)^

Funding ECMO in Brazil is still a challenge. The first report of *Comissão Nacional de Incorporação de Tecnologias no Sistema Único de Saúde* - CONITEC (2021), which is the department of the Ministry of Health in Brazil responsible for incorporating new technologies, had an unfavorable preliminary recommendation for the incorporation of ECMO to support patients with severe acute respiratory syndrome resulting from viral infections refractory to conventional mechanical ventilation in public hospitals.^(28)^ Unfortunately, the latest CONITEC review that occurred in the middle of the COVID-19 pandemic in 2021 still did not recommend ECMO as rescue therapy for adult or pediatric patients with refractory respiratory failure, although more than 100 patients in Brazil had already undergone ECMO for COVID-19 pneumonia (private communication from the Brazilian Chapter of ELSO). Consequently, the SUS would not pay for it. Nonprofit hospitals and private health insurance are still struggling to financially support ECMO, and many patients must pay for the therapy, although recently it has become more common to have ECMO costs covered by private health insurance or by the hospital’s overall budget when the institution has a protocol and the indication is well documented. With this payment approach, ECMO may not be universally available, jeopardizing access for lower economic classes lacking private health insurance.

According to our results, we can say that approximately 15 to 20% of our sample has sufficient knowledge about ECMO management. These physicians responded to the optative clinical cases and specific questions, and we had baseline access to their knowledge.

In the 2 clinical scenarios, 92% of respondents would indicate ECMO as rescue therapy for respiratory failure, and 79% would do so for cardiogenic shock. We added 6 optional questions that were very technical to ascertain the subject’s knowledge about the day-to-day management of patients on ECMO (Table 2). Only 65 participants (15.4%) responded to this segment of the survey. Correct answers ranged from as low as 32.8% to as high as 91.9%, perhaps denoting the level of training and experience of the sample. We can conclude that this tier of respondents has fair/good knowledge of ECMO management, denoting a reasonable level of self-reported ECMO training from these subjects. There is room for improvement in training, especially when most self-reported “experienced” clinicians have managed fewer than 5 patients on ECMO in their careers.

This study has some limitations. Although we surveyed intensivists in different states of the country proportionally to their population, it was not a randomized sample, and it may not reflect the true reality of Brazilian intensivists’ knowledge of ECMO in all parts of the country. Consulted experts felt that we should have included all ECMO centers in Brazil a priori to better represent the true reality of the country. We opted against this approach since there are many more intensivists in the country working in non-ECMO centers, and the ECMO enthusiasts could have biased the final sample. One of the study’s strengths is the large sample size, which is uncommon for a multicenter study in pediatric intensive care in Brazil.

Finally, this study may offer some help on health care policies and planning and may serve as a guide for the application of public resources. We believe it can inspire further research and educational initiatives to educate physicians and rescue more critically ill children with this well-recognized support modality when properly indicated. Better knowledge could also support the establishment of a network of well-prepared referral centers in this vast country of Brazil.

## CONCLUSION

Most Brazilian pediatric intensivists have limited knowledge regarding extracorporeal membrane oxygenation, including its indications and, mainly, its complications. Extracorporeal membrane oxygenation is not yet widely available in Brazilian hospitals, and it is not publicly funded. Very few intensivists are prepared enough to manage extracorporeal membrane oxygenation patients, and most concerning, even fewer intensivists can recognize when to refer patients to extracorporeal membrane oxygenation centers.
